# Cabbage and Sauerkraut Consumption in Adolescence and Adulthood and Breast Cancer Risk among US-Resident Polish Migrant Women

**DOI:** 10.3390/ijerph182010795

**Published:** 2021-10-14

**Authors:** Dorothy Rybaczyk Pathak, Aryeh D. Stein, Jian-Ping He, Mary M. Noel, Larry Hembroff, Dorothy A. Nelson, Fawn Vigneau, Tiefu Shen, Laura J. Scott, Jadwiga Charzewska, Bożena Wajszczyk, Karen Clark, Leszek A. Rybaczyk, Bogdan A. Pathak, Dorota Błaszczyk, Ann Bankowski, Walter C. Willett

**Affiliations:** 1 Department of Epidemiology and Biostatistics, Michigan State University, East Lansing, MI 48824, USA; 2 Hubert Department of Global Health, Rollins School of Public Health, Emory University, Atlanta, GA 30322, USA; aryeh.stein@emory.edu; 3 Department of Epidemiology, Michigan State University, East Lansing, MI 48824, USA; pinghe9@gmail.com (J.-P.H.); bapatha@gmail.com (B.A.P.); dorotabplus@yahoo.com (D.B.); 4 Department of Family Medicine, College of Human Medicine, Michigan State University, East Lansing, MI 48824, USA; noel@msu.edu; 5 Office for Survey Research, Institute for Public Policy and Social Research, Michigan State University, East Lansing 48824, USA; larry.hembroff@gmail.com (L.H.); clarkk@msu.edu (K.C.); 6 Department of Sociology, Anthropology, Social Work and Criminal Justice, Oakland University, Rochester, MI 48309, USA; danelson@oakland.edu; 7 Barbara Ann Karmanos Cancer Institute, Wayne State University, Detroit, MI 48201, USA; vigneauf@med.wayne.edu (F.V.); ann.bankowski@gmail.com (A.B.); 8 Department of Oncology, School of Medicine, Wayne State University, Detroit, MI 48201, USA; 9 Office of Policy, Planning and Statistics, Illinois Department of Public Health, Springfield, IL 62761, USA; Tiefu.Shen@Illinois.gov; 10 Department of Biostatistics, Center for Statistical Genetics, University of Michigan, Ann Arbor, MI 48109, USA; ljst@umich.edu; 11 National Institute of Public Health NIH—National Research Institute, 00-791 Warsaw, Poland; jcharzewska@o2.pl (J.C.); b-wajszczyk@wp.pl (B.W.); 12 Center for Molecular and Human Genetics, The Research Institute at Nationwide Children’s Hospital, Columbus, OH 43205, USA; dorothyrpathak@gmail.com; 13 Department of Nutrition, Harvard T.H. Chan School of Public Health, Boston, MA 02115, USA; wwillett@hsph.harvard.edu; 14 Department of Epidemiology, Harvard T.H. Chan School of Public Health, Boston, MA 02115, USA; 15 Channing Division of Network Medicine, Department of Medicine, Brigham and Women’s Hospital, Harvard Medical School, Boston, MA 02115, USA

**Keywords:** breast cancer, cruciferous vegetables, cabbage, sauerkraut, diet, adolescent diet, adulthood, migrant population

## Abstract

Background: Breast cancer (BC) incidence and mortality are lower in Poland than in the United States (US). However, Polish-born migrant women to US approach the higher BC mortality rates of US women. We evaluated the association between consumption of cabbage/sauerkraut foods and BC risk in Polish-born migrants to US. Methods: We conducted a case–control study of BC among Polish-born migrants in Cook County and the Detroit Metropolitan Area. Cases (*n* = 131) were 20–79 years old with histological/cytological confirmation of invasive BC. Population-based controls (*n* = 284) were frequency matched to cases on age and residence. Food frequency questionnaires assessed diet during adulthood and age 12–13 years. Odds ratios (OR) and 95% confidence intervals (95% CI) were estimated with conditional logistic regression. Consumption of total, raw/short-cooked, and long-cooked cabbage/sauerkraut foods was categorized as low, medium, or high (frequency of servings/week). Results: Higher consumption of total and raw/short-cooked cabbage/sauerkraut foods, during both adolescence and adulthood, was associated with a significantly lower BC risk. Consumption of long-cooked cabbage/sauerkraut foods was low and not significantly associated with risk. The multivariate OR for total cabbage/sauerkraut consumption, high vs. low (>4 vs. ≤2 servings/week) during adolescence was 0.36 (95% CI = 0.18–0.71, p_trend_ < 0.01) and 0.50 (95% CI = 0.23–1.06, p_trend_ = 0.08) during adulthood. For raw/short-cooked cabbage/sauerkraut (>3 vs. ≤1.5 servings/week), the ORs were 0.35 (95% CI = 0.16–0.72, p_trend_ < 0.01) during adolescence and 0.37 (95% CI = 0.17–0.78, p_trend_ < 0.01) during adulthood. For joint adolescent/adult consumption of raw/short-cooked cabbage/sauerkraut foods, (high, high) vs. (low, low), the OR was 0.23 (95% CI = 0.07–0.65). The significant association for high adolescent consumption of raw/short-cooked cabbage/sauerkraut foods and reduced BC risk was consistent across all levels of consumption in adulthood. Conclusion: Greater consumption of total and raw/short-cooked cabbage/sauerkraut foods either during adolescence or adulthood was associated with significantly reduced BC risk among Polish migrant women. These findings contribute to the growing literature suggesting a protective effect of a potentially modifiable factor, cruciferous vegetable intake, on breast cancer risk.

## 1. Introduction

Diet, a modifiable lifestyle factor throughout life, has received much attention in BC research [1]. The relationship between total adult vegetable consumption and risk of BC has been inconsistent [2,3,4,5,6,7,8,9,10,11,12], although an inverse association with risk of estrogen receptor-negative cases has been documented [8,9,11,12]. Less attention has been given to specific vegetables, despite the fact that bioactive phytochemical compound composition of specific vegetables varies greatly [5,9,12].

Cruciferous vegetables, such as broccoli, cabbage, cauliflower, Brussels sprouts, and chemical compounds that are formed at the time of chewing or processing these vegetables, such as indole-3-carbinol (I3C), its digestive derivative 3,3’-diindolylmethane (DIM), and isothiocyanates (ITC) [13], have been evaluated for their cancer preventive properties at various cancer sites [14,15,16,17,18,19]. The anti-carcinogenic mechanisms of these phytochemicals have been studied extensively in vitro and in vivo animal studies [15,20,21,22,23,24,25]. However, the concentration of these compounds and their bioavailability at the time of consumption varies by type of cruciferous vegetable, cultivation conditions, storage, and the method of preparation [26,27,28,29]. In particular, a long cooking duration can lead to a major loss of bioactive compounds, and thus associations with cancer in epidemiological studies may be obscured, if cooking duration is not considered [26].

Lack of accounting for the dose of bioactive compounds may be contributing to the inconsistency reported in epidemiologic literature relating cruciferous vegetable intake to BC risk [5,9,12,30,31,32,33]. However, analyses based on a 30-year follow-up in the Nurses’ Health Study observed a significantly lower (10%) BC risk for high consumption of cruciferous vegetables as a group (>5 vs. ≤2 servings/week). The inverse association remained when intakes of cabbage, broccoli, and cauliflower were evaluated individually [12]. Similarly, a meta-analysis of 11 case–control and 2 cohort studies reported a statistically significant 15% lower breast cancer risk for high cruciferous vegetable intake [33]. 

Annual per capita consumption of cabbage in Poland was and continues be approximately 30 lb, compared with the much lower consumption of 10 lb in the US [34,35,36]. Therefore, Polish-born populations are likely to have a wider range of intakes of cabbage foods during adolescence in Poland as well as in adulthood if they retain their food preferences after migration to the US.

Literature is accumulating which suggest that approaches to BC prevention should also include a focus on dietary exposure during adolescence. This is based on studies of radiation exposure, growth patterns, and adolescent and early adulthood diet that point to adolescence, when breast tissue develops, as a window of susceptibility to BC initiation [37,38,39,40,41,42,43,44,45,46,47].

To summarize, plausible reasons for the inconsistency of current findings between cruciferous vegetable consumption and BC risk include (1) low intakes of cruciferous vegetables in the studied population, (2) failure to account for differences in bioavailability of compounds due to processing and cooking practices, and (3) the focus mainly on adult consumption.

To address these limitations, we conducted a Polish Women’s Health Study (PWHS), a population-based case–control study of BC risk and dietary patterns during adolescence and adulthood, with a focus on levels of consumption of variously prepared cabbage foods in US-resident Polish migrant women, a population with a wide range of cabbage foods consumption.

## 2. Methods

The current study was carried out in Cook County, Illinois and in the Detroit Metropolitan Area (DMA), Michigan, areas with a high concentration of Polish migrants and with population-based cancer registries that allowed for identification of Polish-born BC cases.

All methods followed established protocols approved by the Michigan State University (MSU), Wayne State University (WSU), and the Illinois State Cancer Registry (ISCR) Institutional Review Boards (IRBs), and all interviewed women provided written informed consent.

### 2.1. Study Population

#### 2.1.1. Controls

Recruitment of controls was conducted by the Office for Survey Research (OSR) within the Institute for Public Policy and Social Research (IPPSR) at MSU. For both Cook County and DMA, women aged 20–79 years were identified through population-based Random Digit Dialing (RDD) between 7/1999–7/2002. Controls aged 65–79 were additionally identified through the US Health Care Financing Administration (HCFA). Controls were frequency-matched to cases on site of study in 5-year age intervals. Women were ineligible if they had a previous diagnosis of any cancer other than skin cancer, resided outside the catchment area, or their age was outside the range of 20–79 years. The detail of the RDD and HCFA procedures are provided in Supplemental File 1—Detailed Methods of Control and Case Recruitment and Disposition.

For Cook County, the information on the 358 identified controls (Supplementary Tables S1 and S2) was sent to the National Opinion Research Center (NORC) at the University of Chicago, who was responsible for conducting all interviews in Cook County. Face-to-face interviews were completed by 233 controls, of whom 6 disclosed during the interview that they have been previously diagnosed with BC and were moved from the control to case sample, leaving 227 controls for Cook County (Supplemental Table S2). Information on the 109 eligible controls in DMA (Supplemental Tables S1 and S2) was sent to the Barbara Ann Karmanos Cancer Institute, where the interviewer, trained by NORC was employed. In DMA 76 controls completed face-to-face interviews. One control disclosed during the interview that she has been previously diagnosed with BC and was moved from the control to case sample, leaving 75 controls for DMA. 

Response Rates for Controls: We use Response Rate 4 (RR4), which is based on the American Association for Public Opinion Research (AAPOR) standard definitions [48]. The numerator includes complete and partially complete interviews, and the denominator includes all eligible controls plus an estimated proportion of controls of unknown eligibility, based on the proportion of eligible units in the sample for which a definitive determination of status was obtained. 

The control response rates (RR4) are 74.0% for Cook County and 74.7% for DMA [48] (Supplemental Table S2).

#### 2.1.2. Cases

Eligible cases were defined as Polish-born women with histologically or cytologically confirmed incident invasive BC, who were between 20–79 years old and resident in the study catchment area at the time of diagnosis (Cook County 1/1994–6/2002 and DMA 1/1994–12/2001). Detail of the screening process to identify Polish-born cases is provided in Supplemental Material (Supplemental File 1—Methods of Control and Case Identification and Disposition and Supplemental Table S3).

In Cook County, age-eligible incident BC cases were identified through the Illinois State Cancer Registry (ISCR). Of the 3341 records screened, 266 women were confirmed to be Polish-born, of whom 139 were eligible and 113 agreed to participate. Their information was sent to NORC at the University of Chicago. A total of 105 women completed interviews. An additional 11 cases were identified and interviewed (6 that disclosed at interview that they had previous BC diagnosis and 5 at late recruitment), for a total of 116 cases for Cook County (Supplemental Table S3). In DMA, cases were identified through the Metropolitan Detroit Cancer Surveillance System (MDCSS). Of the 20,721 cases identified for screening, 62 were identified as potentially Polish-born with definite assignment for 57, who were invited to participate, with 36 completing the home interview (includes one case who was initially a control (Supplemental Table S3). Interviewing was conducted by an interviewer residing in the DMA.

Response rates for cases: The RR4 for Cook County is 76.6%, and for DMA, RR4 is 57.1% [48] (Supplemental Table S3).

### 2.2. Exposure Assessment

It has been observed in the literature that while breast cancer incidence and mortality in Poland are much lower than in the US, when Polish women migrate to US, their mortality from BC approaches that of US women [49]. The goal of the PWHS was to evaluate the effect of a traditional Polish diet, which is high in cabbage/sauerkraut foods, on BC risk among Polish migrant women residing in the US. Since the fall of communism in 1989 brought on intense political, social, and economic transformation in Poland and ushered in many dietary changes, the time period of 1985–1989, before the fall of communism, was chosen as the relevant time to assess traditional usual adult diet and other lifestyle exposures. To help participants with the recall of their usual diet and other exposures during this time period, thus reducing recall bias, a life event calendar was used which allowed women to recall important life events, such as marriages, births, change of residence, and major world events that occurred during 1985–1989 [50]. For example, the date of the Chernobyl accident, 4/26/1986 was written in the calendar. For the two calendar years when participants were 12–13 years old, a similar approach was used to orient the women to the calendar time. Participants were asked to remember important life events, such as place of residence (for some this was during WWII) or name of school they attended, or any other event that women considered important that occurred during those two calendar years.

Interviews were conducted between 2000 and 2003. Cases and controls were interviewed at home by trained interviewers using a structured questionnaire. Ten percent of interviews were recorded and reviewed for quality control and feedback to the interviewers.

#### 2.2.1. Dietary Habits

We ascertained usual dietary intake at two reference time points, namely 1985–1989, representing usual adult diet, and when participants were 12–13 years old. The food frequency questionnaire (FFQ) was modeled on the Nurses’ Health Study (NHS) 1986 long questionnaire and Nurses’ Health Study II 1998 Diet questionnaire, and was supplemented with additional detail on cabbage products and other Polish foods, traditionally consumed in Poland, which would be available in Polish delicatessens in the US during 1985–1989 [51]. The final FFQ for 1985–1989 included 143 food items. To ascertain diet in adolescence we used a subset of the full FFQ that ascertained the same information about usual cabbage/sauerkraut foods consumption and was abbreviated for other sections. As the specific question in FFQ was read, participants were also shown graphic cards with the names of products, foods, or drink to aid them with recollection of the frequency of consumption of these foods, products, or drinks during the given time period. Frequency of consumption was assessed in times per day, week, month, or year. For breads, eggs, bacon, and alcoholic beverages, a typical serving size was ascertained. Otherwise, an average serving size was assumed.

Cabbage/sauerkraut consumption (1985–1989, 12–13 years old): FFQ section: “Foods Made from Cabbage,” included questions about nine specific cabbage/sauerkraut foods, traditionally consumed in Poland and the type of cabbage consumed (white/green, savoy, or red cabbage). Total cabbage/sauerkraut consumption was the sum reported servings/week for the individual types of cabbage/sauerkraut foods consumed. Due to differences in the bioavailability of anti-carcinogenic compounds by method of preparation [28,29], total cabbage/sauerkraut food consumption was subdivided into raw/short-cooked and long-cooked cabbage/sauerkraut foods. Raw included salads from raw sauerkraut and fresh cabbage/coleslaw, and short-cooked included boiled sauerkraut and steamed fresh cabbage; long-cooked was the consumption of hunter’s stew (bigos), cabbage rolls (golabki), and as one type of long-cooked cabbage foods, pierogi (dumplings with cabbage), naleśniki (pancakes), or łazanki (pasta) with cabbage, respectively. Based on research on diet of women in the 1980’s, carried out at the Department of Nutritional Epidemiology of the Institute of Food and Nutrition in Warsaw, and in consultation with the Central Statistical Office, GUS, Warsaw, the average serving size of raw salads (sauerkraut or fresh cabbage) was estimated to be 95 grams, and for short-cooked (sauerkraut or fresh cabbage) 60 grams. For long-cooked, average serving size of hunter’s stew (bigos) was estimated at 200 grams, cabbage rolls (golabki) 150 grams, and for the category which combined pierogi, nalesniki and lazanki, average serving size was estimated to be 330 grams. Therefore, by adding the serving sizes for foods consumed in each category, the average serving size for raw/short-cooked foods was 75 grams, and for long-cooked, 225 grams. 

Alcohol consumption (1985–1989): Intakes of beer, wine, and hard liquor during 1985–1989 were assessed, and subsequently calculated as weekly number of servings. Participants were asked how many bottles or cans of beer, or 4 oz glasses of wine, or 2 oz shots of hard liquor they had each time. Total number of weekly servings was calculated as the sum of the weekly servings for each individual alcohol type.

Caloric intake (1985–1989): Total caloric intake was calculated for adult diet. The 1986 Nurses’ Health Study nutrient database was used for caloric intake calculations for US foods, supplemented by data from the Polish food composition database for Polish foods available in Polish delicatessens [52,53].

#### 2.2.2. Other Variables

Reproductive History: Age at menarche was assessed by the onset of first menstrual period. Menopausal status was defined relative to age at diagnosis for cases and age at interview for controls. Women who reported having menstrual cycles were considered to be pre-menopausal; those who provided age at natural menopause were considered post-menopausal; cases who reported that their menstrual cycles stopped due to treatment were considered pre-menopausal; women who had a hysterectomy without removal of ovaries were considered pre-menopausal if their age was less than 50, and postmenopausal if they were 50 years or older, and their age at menopause was assigned to be 50. Age at menopause was taken as reported by the participant. Women who were over 50 at the time of the interview who reported that they had stopped menstruating and did not report their age at menopause were considered to have reached menopause at age 50. Full-term pregnancy (FTP) was defined as a pregnancy with gestational age of more than 24 weeks, irrespective of the outcome. Age at any FTP was calculated from the date when that pregnancy ended relative to the participant’s birthdate. Parity was assessed by the total number of FTP’s, irrespective of outcome.

Oral contraceptive (OC) and hormone replacement therapy (HRT) use: Participants were provided a list of hormonal preparations available as a pill or injections for birth controls. If any of those or other preparations were used for birth control, time period and duration for each use was recorded. Similarly, for other hormone use, participants were provided a list of hormonal preparation pills, patches, or creams, commonly used for HRT as well as for other conditions such as prevention of osteoporosis, regulation of periods, or prevention of miscarriage. The type of each hormone preparation, reason for use, and duration of use were recorded.

Physical activity (1985–1989): Questions were asked about sitting, reclining, and household activities such as sweeping, cooking, doing laundry, heavy cleaning and scrubbing, gardening and other moderate to heavy outside chores, stair climbing and sleeping, and recreational activities (e.g., recreational sports, walking, bicycling, aerobic exercise as well as job activity). Intensity of activity was expressed as METs (Metabolic Equivalent), which were extracted from the Compendium of Physical Activities [54]. Derived daily hours for each activity were multiplied by their respective MET values to create MET h for each activity. Occupational MET hours were calculated using the percentage of work time reported spent on sitting, standing, walking without lifting, walking with lifting <25lbs, and heavy physical work. To account for over/under reporting of total daily hours, a fraction was calculated (hours reported/24). The above fraction was then used to standardize the number of reported daily hours and number of reported daily MET h to a 24-hour day to obtain a total METs/24 hr day.

Family history: We obtained detailed family history of breast and other cancers for first-degree as well as second- and third-degree female relatives on mother’s and father’s side. We considered first-degree family history to be positive if a mother, sister, or daughter had been diagnosed with BC.

Anthropometry: Women reported their maximal height and their weight in each decade of life. We computed 1985–1989 body mass index (kg/m^2^) using height and the weight reported for that time period. For the 13 women missing that weight, we imputed weight as the mean of women within their age decade and case/control status.

Duration of residence in the US by 1985: Women provided information on the year of emigration from Poland and arrival in the US. Women who arrived in the US after 1985 had migration status categorized as “in Poland”, those that arrived before 1985 were categorized as being in the US fewer than 10 years or greater than 10 years prior to 1985.

Highest level of education attained by 1998. Level of education is correlated with type of occupation and is often considered a proxy for socioeconomic status (SES). Therefore, we included in our questionnaire a question which had 9 options for level of education attained, and asked participants to choose the highest level attained by 1989. For these analysis, we grouped them into three levels: Level I—included: no formal schooling (1), elementary school not completed (2), completed elementary school (3), and some (high school (HS) or technical training) (4); Level II—included: completed HS, technicum or gymnasium (5), and vocational, technical, or business training after HS (6); Level III—included: some college/university (7), graduated from college/university (8), and postgraduate or professional school (9).

### 2.3. Statistical Analyses

All analyses were performed using SAS version 9.4 (SAS Inc. Cary, NC, USA). To ensure that the age of participants in 1985–1989 did not overlap with age of 12–13 years, we had to delete 18 controls (from 302 in the original data set—Supplemental Table S2) who were less than 18 years of age in 1989. Therefore, the analyses for this paper are based on 284 controls and 131 cases (Supplemental Table S3).

Descriptive analyses were performed using cross tabulations. Group differences between case and control for categorical variables, were tested in conditional logistic regression models within the joint strata of age and site; age at diagnosis (cases) or interview (controls) (<35 y; 35–44 y; 45–54 y; 55–64 y; 65–74 y; ≥ 75 y) and site (Cook County, DMA); for DMA ages <35 and 35–44 were combined due to small sample size. For the continuous variables of servings/week of total, raw/short-cooked and long-cooked cabbage/sauerkraut foods, PROC GLM was used to obtain p-value for the differences in the least square means adjusted for the joint age and site strata. Conditional logistic regression, using the joint age and site categories as strata was used to estimate the odds ratios (ORs) in models evaluating the association of case status and consumption of cabbage/sauerkraut foods, initially adjusted just for age in 1985 (Model 1) and subsequently adjusted for all confounders (Model 2).

Potential confounders included in the multivariate model were: age in 1985, total energy intake (quartiles), BMI (kg/m^2^) (<18.5, 18.5–<25.0, 25.0–<30, ≥30.0), physical activity (tertiles based on distribution for controls), first degree family history of breast cancer (yes, no), age at menarche (years: <13, 13–<15, ≥15), reproductive history (nulliparous, first full-term pregnancy <22y, 22–<30 y, and ≥30 y), oral contraceptive use (ever, never), hormone replacement therapy use (ever, never), menopausal status at diagnosis (cases) or interview (controls) (premenopausal, postmenopausal), alcohol intake (none, <0.7 servings/week, ≥0.7 servings/week, divided at median number of drinks per week for controls), migration status in 1985 (Poland, in US < 10 years, in US ≥ 10 years), and highest level of education attained by 1989, divided into three levels (levels: I—some HS or below, II—completed HS or other technical or business training after HS, III—some or completed college/university, postgraduate, or professional school).

Conditional logistic regression, evaluating the association of case status and consumption of cabbage/sauerkraut foods, was carried out independently for adolescent and adult consumption. ORs adjusted for age in 1985, (Model 1) and multivariate-adjusted (for all potential confounders) ORs (Model 2), were calculated for total, raw/short-cooked, and long-cooked cabbage/sauerkraut foods consumption, categorized as low (L), medium (M), and high (H). Categories of servings/week were: total consumption [L (≤2), M (>2–≤4), H (>4)], raw/short-cooked [L (≤1.5), M (>1.5–≤3), H (>3)] and long-cooked [L (≤0.5), M (>0.5–≤1), H (>1)]; linear trend was assessed by modeling the median value for each category within each cabbage foods type, as a continuous variable. For all models, p-values were calculated by the Wald test [55].

To evaluate the association of the joint consumption of raw/short-cooked cabbage/sauerkraut foods (the category potentially with the least noise in the bioavailability of anti-carcinogenic compounds and representing on average 70% of total consumption), we created 9 categories of consumption (combinations of the 3 levels, L, M, H, in adolescence and adulthood, 3 × 3) with low consumption of raw/short-cooked cabbage/sauerkraut foods in both adolescence and in adulthood (LL) as the reference category.

## 3. Results

Cases were similar to controls with respect to age (diagnosis for cases, interview for controls), age in 1985, migration status in 1985, age at menarche, oral contraceptive use, hormone replacement therapy and menopausal status (at time of diagnosis for cases and interview for controls), BMI, total energy intake, and alcohol intake during 1985–1989. Cases were more likely to report a first degree relative with BC, were older at first birth, reported lower levels of physical activity, and attained a higher level of education by 1989 (Table 1).

Participants reported that they mainly consumed white/green cabbage. Table 2 provides the distribution of cabbage/sauerkraut foods consumption for cases and controls, both in adolescence and adulthood. Cases had lower consumption for each percentile of the distribution and significantly lower mean consumption for total and raw/short-cooked cabbage/sauerkraut foods.

In conditional logistic regression, with the joint age and site as strata, multivariate adjustment for potential confounders did not change our OR estimates substantially from those observed in Model 1, Table 3. Our multivariate ORs (Model 2, Table 3) are as follows: OR for total high vs. low cabbage/sauerkraut food consumption (>4 vs. ≤2 servings/week) during adolescence is 0.36 (95% CI = 0.18–0.71, p_trend_ < 0.01) and during adulthood OR = 0.50 (95% CI = 0.23–1.06, p_trend_ = 0.08). The multivariate ORs for raw/short-cooked cabbage/sauerkraut foods (>3 vs. ≤1.5 servings/week) is 0.35 (95% CI = 0.16–0.72, p_trend_ ≤ 0.01) during adolescence and 0.37 (95% CI = 0.17–0.78, p_trend_ < 0.01) during adulthood. Consumption of long-cooked cabbage/sauerkraut foods was low and not significantly associated with risk. 

In the model for joint consumption levels of the raw/short-cooked cabbage/sauerkraut foods during adolescence and adulthood, adjusted for all potential confounders, a significantly lower BC risk was observed OR = 0.23 (95% CI = 0.07–0.65) for the comparison of (high, high) vs. (low, low-reference) consumers (high >3 servings/week, low ≤1.5 servings/week) (Table 4). It is worth noting that high consumption in adolescence was associated with a significant reduction in risk (of over 70%) throughout life regardless of consumption levels in adulthood, with ORs ranging between 0.23 to 0.27 (bottom set of cells marked in green). In addition, high consumption of raw/short-cooked cabbage/sauerkraut foods in adulthood, was also associated with significant reduction in risk for those who were low or medium consumers in adolescence and was comparable (over 70% reduction) to the reduced risk attained by high consumers during adolescence (vertical set of right most cell marked in green). 

## 4. Discussion

In this population-based case–control study, in our multivariate models which adjusted for potential confounders, we observed associations of high consumption of cabbage/sauerkraut foods in adolescence and in adulthood with reduced BC risk. Specifically, we observed a dose–response relationship between increased consumption of total and raw/short-cooked cabbage/sauerkraut products and decreased BC risk, with no reduction in risk observed for long-cooked cabbage/sauerkraut products. We also observed a significant association between high consumption of raw/short-cooked cabbage/sauerkraut foods, jointly during both adolescence and adulthood, and reduced BC risk relative to low consumption during both time periods. Further, when evaluating the effect of joint consumption levels during the two time periods, the observed association between high adolescent consumption of raw/short-cooked cabbage/sauerkraut foods and reduced BC risk was not influenced by level of consumption during adulthood. Similarly, among women with low or medium consumption during adolescence, high consumption in adulthood was associated with a lower risk, similar to that among women with high consumption in adolescence.

Cabbage and other brassica vegetables are a rich source of glucosinolates. While in an intact plant, glucosinolates and the enzyme myrosinase remain in separate sub-cellular compartments; upon cutting or chewing, myrosinase converts glucosinolates into a variety of bioactive compounds such as indole-3-carbinol (I3C) and isothiocyanates (ITC). Cabbage contains high levels of indole glucosinolates that form I3C, in contrast to broccoli where aliphatic glucosinolates predominate and form ITC [28,56]. The indoles undergo further metabolism via acid-catalyzed reactions occurring in the low pH environment of the stomach to produce several compounds, including the condensation product 3,3′-diindolylmethane (DIM) [23].

However, formation of these compounds depends on the method of preparation. Cooking denatures myrosinase, and a portion of intact glucosinolates is degraded or leached from the vegetable tissue. Estimates of the metabolically available bioactive compounds, depending on duration and type of cooking, range from 80% for short-cooked (10 min or less), down to 7% for long-cooked [27,28,29].

The findings of a simulation study that hypothesized a significant reduction in cancer risk with increasing cruciferous vegetable consumption, when all sources of variation in bioavailability were able to be accounted for (i.e., cultivation, storage, and processing (cooking)), influenced our approach to separately evaluate long vs. raw/short-cooked cabbage/sauerkraut food consumption and BC risk [26]. In that study, when sources of variation were not accounted for in the model, no association between level of consumption and cancer risk was observed. In contrast, accounting for methods of preparation allowed the model to detect, in the same data set, a lower than originally hypothesized yet significant association between cruciferous vegetable consumption and reduced cancer risk. These results, when combined with ours, suggest that a review of existing studies of cruciferous vegetables and BC risk could be warranted if their data collection would allow for adjustment of cruciferous vegetable consumption by method of preparation.

Carcinogenesis is a multistep and multi-pathway process with phases of initiation, promotion and progression [57]. In vitro and in vivo animal studies evaluating the anti-carcinogenic properties of I3C, DIM and ITC, have shown that they: decrease cell proliferation, induce apoptosis, induce Phase II enzymes which are involved in detoxification of carcinogens, downregulate the expression of the estrogen-responsive genes, induce genes involved in DNA repair, and induce genes that can shift estrogen metabolism to favor hydroxylation of 17β-estradiol to less estrogenic forms [15,23], thus blocking the processes that lead to cells’ acquisition of the hallmarks of cancer [58].

In animal studies, Wattenburg and Loub showed that oral intubation of I3C in a single dose prior to carcinogen treatment reduced the incidence and multiplicity of DMBA-induced mammary tumors in rats by up to 80% [24]. These observations were confirmed by Grubbs et.al [25]. Our findings of a significantly lower risk of breast cancer associated with high consumption of raw/short-cooked cabbage/sauerkraut foods in adolescence parallel these findings, and suggest that these compounds can play a role in reducing cancer occurrence by reducing the rate of initiation. Animal studies also provide support for a protective effect for consumption of these foods during adulthood. Chen et al. showed that repeated, oral administration of DIM during the promotion stage of DMBA-induced mammary tumors reduced their growth by as much as 95% [59]. For a compound to be an anti-promoter, it must be given repeatedly, at a threshold dose, over an extended period of time after a potential carcinogen exposure (and initiation). Our finding of protection offered by high consumption (representing the threshold dose) of the raw/short-cooked cabbage/sauerkraut foods during adulthood by women who were low or medium consumers in adolescence points to a role these compounds can play in the promotion stage of carcinogenesis. 

In a case–control study of Asian-Americans in Los Angeles County, with over 70% of study participants being non-US born, a high intake of soy during adolescence was associated with reduced BC risk, with high adult consumption showing weaker association [40]. Analyzing the joint consumption of soy during adolescence and adulthood, authors observed that individuals who were high soy consumers during both time periods showed the lowest risk (OR = 0.53. 95% CI = 0.36–0.78) compared with those who were low soy consumers during both time periods. Our findings, for the protective effect of high cabbage/sauerkraut product consumption, parallel those observed for soy consumption in Asian populations. We observed that high consumption, especially of raw/short-cooked cabbage/sauerkraut products, was associated with a significant reduction in BC risk both during adolescence and adulthood. We also observed the greatest reduction in BC risk in subjects who were high consumers during both adolescence and adulthood, relative to those who were low consumers in both time periods (OR = 0.23, 95% CI = 0.07–065). In both case–control studies, when consumption of culturally specific foods containing phytochemicals hypothesized to have anti-carcinogenic properties (though potentially by different mechanisms of action) is evaluated, a similar pattern of association between a high consumption of these foods and reduced breast cancer risk has been observed. One of the potentially contributing factors in the observed increase in breast cancer risk for populations migrating from low-risk for breast cancer incidence/mortality countries to the high-risk countries is the potential reduction in consumption of culturally specific foods which have anti-carcinogenic properties and are consumed at high levels in the countries of origin.

Strengths of our study include: the uniqueness of our population—to date no other study has been conducted in this population; ascertainment of population-based cases through cancer registries and controls by random digit dialing, supplemented with information from HCFA for potential controls aged 65–79 years old; detailed assessment of cabbage and sauerkraut consumption at two key points in a woman’s life, namely adolescence and adulthood; and consideration of the type of food preparation and the potential for loss of bioavailability and potency.

There are also several limitations. First, due to the relatively small sample size our observed results need to be interpreted with caution. The small sample size also limited our power to assess the heterogeneity of associations across potential effect modifiers such as family history, BMI, menopausal status, or attained educational level by 1989. Second, as in any case–control study, diet recall was retrospective and thus potential for recall bias cannot be excluded. Since the data were collected in the early 2000s, when there was less knowledge among the general population about cruciferous vegetables and cancer, this potentially reduced recall bias. Even though, for the adolescent diet in particular, women were asked to recall a period that was up to 65 years prior to interview, for many of them this coincided with World War II or the early 1950s, salient periods when food shortages were common in Poland. For the period 1985–1989, women could identify with the Solidarity movement in Poland and the 1986 Chernobyl atomic reactor accident. Use of the life events calendar helped participants to gain better access to long-term memory for those time periods. Use of show cards helped participants evoke memories about consumption of specific foods/products during those two time periods. We doubt that women in our study were aware of the specific study hypotheses under examination, and hence we do not believe that reporting bias would likely be differential by case/control status—we are unable, of course, to test this assumption. Third, even though almost all of our participants lived within the sampled census tracts (See Supplemental Material: Methods of Control and Case Identification and Disposition), they represent only a small proportion of the population of those census tracts, and hence sample sizes were small. Finally, while our analysis adjusted for many reproductive, biological, and behavioral risk factors for BC, residual confounding cannot be excluded.

## 5. Conclusions

To our knowledge, this is the first study to investigate the associations of high consumption of total and specifically raw/short-cooked cabbage/sauerkraut foods with BC risk during both adolescence and adulthood. Together with findings from other studies (both case–control and cohort), which observed the protective effect of cruciferous vegetables on BC risk, especially the findings based on a 30-year follow-up in the Nurses’ Health Study, these findings have implications for dietary recommendations for consumption of cabbage/sauerkraut foods across a lifetime to potentially reduce the risk of breast cancer. Further studies are needed to corroborate these findings, especially longitudinal studies that will take into account adolescent diet.

## Figures and Tables

**Table 1:** Selected characteristics of Polish-born women residing in Cook County, IL or the Detroit Metropolitan Area, MI, by case–control status.

Variables	Cases (*n* = 131)	Controls (*n* = 284)	*p*^1,^*
	**%**	**%**	
**Study site**			0.7 ^2^
Cook County, Chicago	77.1	76.1	
Detroit Metropolitan Area	22.9	23.9	
**Age (y) at diagnosis (cases)/interview (controls)**			0.3 ^3^
<35	4.6	5.6	
35–44	19.1	14.8	
45–54	30.5	27.8	
55–64	20.6	16.2	
65–74	19.1	23.9	
≥75	6.1	11.6	
**Age (y) in 1985**			0.3 ^3^
<35	36.6	34.5	
35–44	22.9	22.2	
45–54	22.9	25.0	
55–64	13.7	17.6	
≥65	3.8	0.7	
**Migration status in 1985**			0.2
In Poland	43.5	44.4	
In US < 10 y	22.9	25.0	
In US ≥ 10y	33.6	30.6	
**First degree family history of breast cancer**	15.3	8.1	**0.02**
**Age at menarche (y)**			0.3
<13	23.7	20.8	
13–<15	53.4	47.9	
≥15	22.9	31.3	
**Age at first full-term pregnancy (y)**			**0.03**
Nulliparous	11.5	9.2	
<22	20.6	28.5	
22–<30	51.9	53.9	
30+	16.0	8.4	
**Ever used oral contraception**	15.3	12.0	0.4
**Ever used hormonal replacement therapy**	14.5	11.6	0.4
**Premenopausal at diagnosis (cases)/interview (controls)**	44.3	38.4	0.8
**BMI in 1985–1989 (kg/m^2^)**			0.8
<18.5	3.8	5.6	
18.5–<25.0	64.9	59.5	
25.0–<30.0	25.2	26.1	
≥30.0	6.1	8.8	
**Physical activity in 1985–1989 (METS/24 h)**			**0.01**
≤48.8	45.0	33.1	
48.8–<59.6	35.1	33.4	
≥59.6	19.9	33.5	
**Total energy intake in 1985–1989 (kcal/d)**			0.3
<1935	32.8	25.0	
1935–<2365	24.4	25.0	
2365–2880	24.4	25.0	
≥2880	18.3	25.0	
**Alcohol intake in 1985–1989 (drinks/week)**			0.5
none	9.2	14.8	
<0.7	46.2	42.6	
≥0.7	44.6	42.6	
**Highest level of education attained by 1989 (Levels I, II, III)**			**0.001**
I—some high school (HS)/technical training or below	21.4	39.1	
II—Completed HS/Technical or Business training after HS	44.3	42.6	
III—Some or graduated from college, postgraduate or professional school	34.3	18.3	

^1^ Comparison between cases and controls, within the combined strata of age at diagnosis(cases) or interview (controls) (<35 y; 35–44 y; 45–54 y; 55–64 y; 65–74 y; ≥75 y) and site (CC, DMA); For DMA (ages <35 y and 35–44 y) combined due to small sample size. ^2^ Adjusted for age at diagnosis (cases) or interview (controls). ^3^ Adjusted for site. * Bold indicates statistical significance of *p* < 0.05.

**Table 2:** Percentile distribution and least square means (LSM) ^a^, and standard error of the mean (SEM) for cabbage/sauerkraut food consumption (servings/week) during adolescence and adulthood.

Type of Cabbage/ Sauerkraut Food		12–13 Years Old Adolescence Servings/Week			1985–1989 Adulthood Servings/Week		
	Percentile LSMean SEM	Control (284)	Case (131)	Delta Control- Case	Control (284)	Case (131)	Delta Control- Case
Total	75th	4.60	3.67	0.93	4.18	3.72	0.46
	50th	3.08	2.41	0.67	2.96	2.23	0.73
	25th	1.82	1.38	0.44	1.52	1.39	0.13
	LSMean (SEM)	3.38 (0.16)	2.64 (0.22)	0.74 **	3.07 (0.14)	2.49 (0.20)	0.57 **
Raw/short-cooked	75th	3.31	2.40	0.91	2.96	2.46	0.50
	50th	2.05	1.66	0.39	1.82	1.50	0.32
	25th	1.17	0.90	0.27	0.92	0.77	0.15
	LSMean (SEM)	2.44 (0.13)	1.86 (0.18)	0.58 **	2.12 (0.11)	1.62 (0.15)	0.50 **
Long-cooked	75th	1.36	1.13	0.23	1.27	1.23	0.04
	50th	0.69	0.69	0.00	0.76	0.75	0.01
	25th	0.34	0.40	−0.06	0.46	0.40	0.06
	LSMean (SEM)	0.93 (0.06)	0.77 (0.08)	0.16 (**NS**)	0.94 (0.05)	0.88 (0.07)	0.06 (**NS**)

^a^ Least square means (LSMean) and standard error of the mean (SEM) adjusted for age at diagnosis(cases) or interview (controls) (<35 y; 35–44 y; 45–54 y; 55–64 y; 65–74 y; ≥ 75 y) and site (Cook County, DMA). For DMA (ages < 35 y; 35–44 y) were combined due to small sample size; ** bold represents *p* ≤ 0.01; (NS)-not significant.

**Table 3:** Risk of breast cancer in association with intake of cabbage/sauerkraut foods during adolescence or adulthood amongst Polish-born women residing in Cook County, IL or the Detroit Metropolitan Area, MI.

Cabbage/Sauerkraut	Servings Per Week	Number Cases, Controls	Model 1 OR ^a^	95% CI	Trend 1 Serv/Week OR (95% CI) ^b^ *p*-Value	Model 2 OR ^c^	95% CI	Trend 1 Serv/Week OR (95% CI) ^b^ *p*-Value
**At age 12–13**								
**Total**	≤2	58, 86	1.0	(-)	**0.79 (0.68–0.91)** *p* = 0.001	1.0	(-)	**0.79 (0.67–0.92)** *p* = 0.003
	2–≤4	49, 102	0.65	0.38–1.10		0.67	0.37–1.18	
	>4	24, 96	**0.37**	**0.19–0.68**		**0.36**	**0.18–0.71**	
								
**Raw/Short-cooked**	≤1.5	62, 94	1.0	(-)	**0.72 (0.58–0.87)** *p* = 0.001	1.0	(-)	**0.73 (058–0.90)** *p* = 0.004
	1.5–≤3	51, 111	**0.60**	**0.36–1.00**		0.63	0.36–1.09	
	>3	18, 79	**0.33**	**0.16–0.64**		**0.35**	**0.16–0.72**	
**Long-cooked**								
	≤0.5	50, 104	1.0	(-)	0.84 (0.59–1.19) *p* = 0.33	1.0	(-)	0.84 (0.56–1.23) *p* = 0.36
	0.5–≤1	43, 73	1.14	0.64–2.01		1.05	0.56–1.95	
	>1	38, 107	0.80	0.45–1.38		0.78	0.41–1.44	
**In 1985–1989**								
**Total**	≤2	55, 98	1.0	(-)	**0.84 (0.72–0.97)** *p* = 0.02	1.0	(-)	0.84 (0.69–1.02) *p* = 0.08
	2–≤4	52, 107	0.87	0.52–1.45		0.86	0.47–1.56	
	>4	24, 79	**0.48**	**0.25–0.87**		0.50	0.23–1.06	
**Raw/Short-cooked**	≤1.5	67, 118	1.0	(-)	**0.75 (0.61–0.92)** *p* = 0.005	1.0	(-)	**0.73 (0.57–0.93)** *p* = 0.009
	1.5–≤3	45, 99	0.74	0.44–1.23		0.70	0.38–1.27	
	>3	19, 67	**0.40**	**0.20–0.75**		**0.37**	**0.17–0.78**	
**Long-cooked**	≤0.5	43, 77	1.0	(-)	0.87 (0.57–1.31) *p* = 0.50	1.0	(-)	1.03 (0.62–1.70) *p* = 0.92
	0.5–≤1	44, 107	0.70	0.39–1.22		0.73	0.39–1.37	
	>1	44, 100	0.77	0.43–1.35		0.96	0.48–1.90	

**^a^** OR adjusted for age in 1985 within the combined strata of age at diagnosis(cases) or interview (controls) (<35 y; 35–44 y; 45–54 y; 55–64 y; 65–74 y; ≥75 y) and site (Cook County, DMA). For DMA (ages <35 y and 35–44 y) combined due to small sample size. ^b^ Linear trend on median values for categories, modeled as a continuous variable ^c^ Additionally, adjusted for BMI in 1985–1989 (<18.5 kg/m^2^; 18.5–24.99 kg/m^2^; 25.0–29.99 kg/m^2^; ≥30.0 kg/m^2^), physical activity in 1985–1989 (quartiles), total energy intake in 1985–1989, family history of breast cancer (yes; no), age at menarche (<13 y; 13–14 y; ≥15 y), reproductive history (nulliparous; first full-term pregnancy < 22 y; first full-term pregnancy 22–29 y; first full-term pregnancy ≥ 30 y), oral contraceptive use (ever; never), hormone replacement therapy use (ever; never), menopausal status at diagnosis (cases) or interview (controls) (premenopausal; postmenopausal), alcohol intake in 1985–1989 (none; <0.7 serving/week; ≥0.7 serving/week), migration status in 1985 (Poland; in US < 10 y; in US ≥ 10 y) and highest level of attained education by 1989 ( levels: I—some HS or below, II—completed HS or other technical or business training after HS, III—some or completed college/university, postgraduate or professional school). Bold, represents OR’s that are statistically significant at *p* < 0.05.

**Table 4:** Breast cancer risk in Polish-born women residing in Cook County, IL or the Detroit Metropolitan Area, MI: Adjusted ORs for joint levels of consumption of raw/short-cooked cabbage/sauerkraut products during adolescence and adulthood.

**Cell Legend** **Top:** OR ^a^; bold: *p* < 0.05 **Middle:** 95% CI **Bottom: No**. Cases, No. Controls		**Adult** Servings per week		
		Low ≤1.5/wk	Medium 1.5–≤3.0/wk	High >3.0/wk
**Adolescent** Servings per week	Low ≤1.5/wk	1.0	0.57	0.29
		(Reference)	0.21–1.51	0.07–1.14
		48, 58	14, 24	4, 12
	Medium 1.5–≤3.0/wk	0.51	0.54	**0.27**
		0.23–1.10	0.24–1.19	0.08–0.83
		20, 44	24, 46	7, 21
	High >3.0/wk	0.27	**0.27**	**0.23**
		0.06–1.14	0.08–0.86	0.07–0.65
		3, 16	7, 29	8, 34

^a^ Within the combined strata of age at diagnosis(cases) or interview (controls) (<35 y; 35–44 y; 45–54 y; 55–64 y; 65–74 y; ≥75 y) and site (Cook County, DMA; for DMA—ages <35 y and 35–44 y combined due to small sample size), adjusted for age in 1985, BMI in 1985–1989 (<18.5 kg/m^2^; 18.5–24.99 kg/m^2^; 25.0–29.99 kg/m^2^; ≥30.0 kg/m^2^), physical activity in 1985–1989 (quartiles), total energy intake in 1985–1989 (quartiles), family history of breast cancer (yes; no), age at menarche (<13 y; 13–14 y; ≥15 y), reproductive history (nulliparous; first full-term pregnancy < 22 y; first full-term pregnancy 22–29 y; first full-term pregnancy ≥ 30 y), oral contraceptive use (ever; never), hormone replacement therapy use (ever; never), menopausal status at diagnosis (cases) or interview (controls) (premenopausal; postmenopausal), alcohol intake in 1985–1989 (none; <0.7 serving/week; ≥0.7 serving/week), migration status in 1985 (in Poland; in US < 10 y; in US ≥ 10 y), and highest level of attained education by 1989 (levels: I—some HS or below, II—completed HS or other technical or business training after HS, III—some or completed college/university, postgraduate or professional school). Bold, represents OR’s that are statistically significant at *p* < 0.05.

## Data Availability

The dataset used for the analysis of the current study is available from the corresponding author on reasonable request.

## Supplementary Materials

The following are available online at https://www.mdpi.com/article/10.3390/ijerph182010795/s1, Methods of Control and Case Identification and Disposition, Table S1: Dual Mode Recruitment of Controls Using Population Based Samples and Health Care Finance Administration (HCFA) Listings for Cook County, IL and Metropolitan Detroit Area, MI, Table S2: Disposition of Identified Controls amongst Polish-born women residing in Cook County IL or in the Detroit Metropolitan Area, MI, Table S3: Identification and Disposition of Cancer Cases amongst Polish-born women residing in Cook County IL or in the Detroit Metropolitan Area, MI.

## Abbreviations

US	United States
BC	Breast cancer
OR	Odds ratio
95% CI	95% confidence interval
I3C	indole-3-carbinol
DIM	3,3′-diindolylmethane
ITC	isothiocyanates
DMA	Detroit Metropolitan Area
MSU	Michigan State University
WSU	Wayne State University
ISCR	Illinois State Cancer Registry
IRB	Institutional Review Board
OSR	Office for Survey Research
IPPSR	Institute for Public Policy and Social Research
RDD	Random Digit Dialing
HCFA	Health Care Financing Administration
NORC	National Opinion Research Center
RR4	Response Rate 4
AAPOR	American Association for Public Opinion Research
PWHS	Polish Women’s Health Study
FFQ	Food frequency questionnaire
NHS	Nurses’ Health Study
MDCSS	Metropolitan Detroit Cancer Surveillance System
FTP	Full-term pregnancy
OC	Oral contraceptive
HRT	Hormone replacement therapy
MET	Metabolic Equivalent
MET-h	MET hours
BMI	Body mass index
DMBA	7,12-dimethylbenz[a]anthracene
