# Supplementary material for: Cabbage and Sauerkraut Consumption in Adolescence and Adulthood and Breast Cancer Risk among US-Resident Polish Migrant Women
Source: Int J Environ Res Public Health. 2021 Oct 14;18(20):10795. doi: 10.3390/ijerph182010795 (PMC8535652; doi:10.3390/ijerph182010795)
Supplement: Supplementary file 1 [file ijerph-18-10795-s001.zip › ijerph-1399260-supplementary.pdf]

Supplementary File: Cabbage and Sauerkraut Consumption in Adolescence and Adulthood and Breast Cancer Risk among US-Resident Polish Migrant Women

Table S1: Dual Mode Recruitment of Controls Using Population Based Samples and Health Care Finance Administration (HCFA) Listings for Cook County, IL and Metropolitan Detroit Area, MI

Table S2: Disposition of Identified Controls amongst Polish-born women residing in Cook County IL or in the Detroit Metropolitan Area, MI

Table S3: Identification and Disposition of Cancer Cases amongst Polish-born women residing in Cook County IL or in the Detroit Metropolitan Area, MI

Detailed Methods of Control and Case Recruitment and Disposition

<u>Study Population</u>

Controls: Recruitment of controls was conducted by the Office for Survey Research (OSR) within the Institute for Public Policy and Social Research (IPPSR) at MSU. Women aged 20-79 were identified through population-based Random Digit Dialing (RDD) between 7/1999-7/2002, with identification of controls aged 65-79, supplemented through the US Health Care Financing Administration (HCFA).

To enhance the efficiency of RDD, census data were used to identify the proportion of Polish-born residing in each census tract within Cook County and the DMA (Wayne, Oakland, and Macomb counties). Tracts were stratified into low, medium and high-density using area-specific thresholds.

In Cook County, low, medium, and high densities were defined as 0.5% -4.99%, 5.0% - 14.99%, and 15% or more Polish-born, respectively. Tracts where the Polish-born population was less than 0.5% were excluded. The total number of Polish-born residents in the 515 sampled tracts represented 95.6% of all Polish-born residents in Cook County and 3.0% of all residents in these tracts. In DMA, low, medium, and high density was defined as 0.2% - 0.99% 1.0% - 4.99%, and 5.0% or more Polish-born. Tracts

with less than 0.2% Polish-born residents were excluded. The total number of Polish-born residents within the 424 sampled tracts represented 94.4% of all Polish-born residents of Wayne, Oakland, and Macomb counties, and 0.83% of all residents in these tracts.

A household was considered potentially eligible for the study if a Polish-born woman between the ages of 20 and 79 was residing there. Within each density stratum, a sample of listed phone numbers was drawn from among households whose addresses fell within the stratum. From the telephone blocks associated with the listed numbers, a second sample was drawn of unlisted phone numbers. Therefore, both listed and unlisted numbers were represented in the sample.

The targeted number of controls for each 5-year age group strata was 1.5 times the number of the projected cases. This allowed for the possibility that recruited controls when invited for the in-person interview, might refuse, be unavailable, or the quota for that age group would already have been met. In households where more than one woman was Polish born, one was chosen at random from among those in the age group(s) where the quota was not yet met.

The calling protocols for the study (for both RDD and HCFA controls with phone numbers available) included up to 12 call attempts, with calls made at various times of day and days of the week. Screening was conducted by bilingual interviewers. Initial refusals were contacted for possible conversion 8 days later. Potential HCFA controls whose address listing could not be matched to a telephone number were sent a letter explaining the purpose of the study, eligibility criteria, and a postage-paid postcard for the woman to complete and return to IPPSR with contact information for further screening.

In Cook County, 29,042 phone numbers were used to identify 429 Polish women (yield 1.5%), of whom 267 were determined to be eligible and agreed to participate in the face-to-face interview (yield 0.9%). From HCFA, 83 additional controls were identified, for a total of 350 (Table S1). Prior to IPPSR involvement in the study, Gordon Research conducted RDD in Cook County using 2451 listings and identified 8 potential controls. Within two months, the firm closed, and IPPSR became responsible for all control recruitment. Thus, with the 8 additional controls identified by Gordon Research 358 controls were identified for Cook County (Table S2). Their information was sent to the

National Opinion Research Center (NORC) at the University of Chicago, who was responsible for conducting all interviews in the Cook County. Six controls disclosed during the interview that they were previously diagnosed with BC (reassigned to a new case ID) and were removed from the control sample for a total of 352 eligible controls (Table S2).

In DMA, a total of 22,820 phone numbers were used to identify 100 potentially eligible women (yield 0.4%), of which 75 were eligible and agreed to participate (yield 0.3%). From HCFA, 34 additional controls were identified, for a total of 109 (Table S1). Their information was sent to Barbara Ann Karmanos Cancer Institute, where the interviewer for the DMA, trained by NORC, was employed. One control disclosed during the interview that she was previously diagnosed with BC (reassigned to a case ID), for a total of 108 eligible controls.

Cases: Case recruitment initially followed a standard protocol common to both registries where a letter was sent to a physician for approval to contact the patient. If denied no contact was initiated with the patient. If approved or no response for 3 weeks (considered passive approval), this was followed by a letter to the potential participant, explaining the study and inviting their participation. For those whose place of birth was unknown, site specific approaches that would maximize identification of potentially Polish-born cases (such as common Polish last or first name, residence zip code where Polish populations reside, etc.) were employed to identify potential Polish-born cases for further screening. All screening approaches were approved by the respective IRB.

In Cook County, age-eligible incident BC cases were identified through the Illinois State Cancer Registry (ISCR). Trained abstractors screened from 51 hospitals a total of 3,341 records, identified as White and born in Poland or with unknown place of birth. Of these, 1,754 (52.5%) were determined to be non-Polish born, 313 (9.4%) were subsequently confirmed to be ineligible, and we were unable to establish eligibility for 1,008 (30.2%). A total of 266 (8.2%) women were confirmed to be Polish-born, of whom 139 were eligible and 113 (81.3%) agreed to participate (Table S3). Their information was sent to NORC, at the University of Chicago; 105 women completed interviews. Six interviewed controls disclosed that they were BC cases during interview (n=111). After NORC's contract with PWHS was finalized, 5 more cases were interviewed by the same interviewer, now employed by MSU (cases n=116).

In DMA, cases were identified through the Metropolitan Detroit Cancer Surveillance System (MDCSS). All age-eligible, living, white or unknown race, female, incident BC cases residing in Macomb, Oakland and Wayne Counties identified with either place of birth in Poland or with unknown place of birth were identified by retrospective data extract and via rapid reporting of new cases. 20,721 cases were identified for screening. Of those, 1757 (8.5%) were ineligible. We identified 62 Polish-born women and the screening process resulted in a definite assignment for 57, who were invited to participate, with 36 completing the home interview (Table S3).

Table S1. Dual Mode Recruitment of Controls Using Population Based Samples and Health Care Finance Administration (HCFA) Listings for Cook County, IL and Metropolitan Detroit Area, MI

	Cook County		Metropolitan Detroit Area		Combined Site Total		Cook County		Metropolitan Detroit Area		Combined Site Total		Total All Sites and Modes	
	Phone Recruitment (Random Digit Dialing)						HCFA Recruitment							
**Phone Recruitment**	N	%	N	%	N	%	N	%	N	%	N	%	N	%
**Total Screened – Phone Recruitment**	**29,042**	**100.0%**	**22,820**	**100.0%**	**51,862**	**100.0%**	**4,462**	**100.0%**	**4,884**	**100.0%**	**9346**	**100.0%**	**61,208**	**100.0**
Incapable (mental/physical impairment)[a]	804	2.8%	241	1.1%	1,045	2.0%	120	2.7%	101	2.1%	221	2.4%	1,266	2.1%
Ineligible or non-household[b]	17,732	61.1%	8,275	36.3%	26,007	50.1%	3,389	76.0%	3,751	76.8%	7,140	76.4%	33,147	54.2%
No contact - calling protocol met[c]	1,630	5.6%	3,523	15.4%	5,153	9.9%	220	4.9%	391	8.0%	611	6.5%	5,764	9.4%
Non-working phone numbers[d]	5,276	18.2%	8,317	36.4%	13,593	26.2%	221	5.0%	196	4.0%	417	4.5%	14,010	22.9%
Quota met[e]	39	0.1%	5	0.0%	44	0.1%	0	0.0%	0	0.0%	0	0.0%	44	0.1%
Refusal[f]	1,789	6.2%	2,239	9.8%	4,028	7.8%	387	8.7%	391	8.0%	778	8.3%	4,806	7.9%
Study calling protocol not met[g]	1,299	4.5%	0	0.4%	1,299	2.5%	0	0.0%	0	0.0%	0	0.0%	1,299	2.1%
Unable to complete screening[h]	44	0.2%	120	0.5%	164	0.3%	10	0.2%	20	0.4%	30	0.3%	194	0.3%
**Screened – met eligibility criteria**	**429**	**1.5%**	**100**	**0.4%**	**529**	**1.0%**	**115**	**2.6%**	**34**	**0.7%**	**149**	**1.6%**	**678**	**1.1%**
**Mail Recruitment**	N	%	N	%	N	%	N	%	N	%	N		N	%
**Total Screened – Mail Recruitment**							**2,471**	**100.0**	**2,187**	**100.0**	**4,658**	**100.0%**	**4,658**	**100.0%**
Ineligible							625	25.3%	555	25.4%	1,180	25.3%	1,180	25.3%
No contact (eligibility unknown)			N/A				1,838	74.4%	1,624	73.3%	3,462	74.3%	3,462	74.3%
**Screened – met eligibility criteria**							**8**	**0.3%**	**8**	**0.4%**	**16**	**0.3%**	**16**	**0.3%**
**Final Screening and Recruitment**	N	%	N	%	N	%	N	%	N	%	N		N	%
**Eligible for Final Screening**	**429**	**100.0%**	**100**	**100.0%**	**529**	**100.0%**	**123**	**100.0%**	**42**	**100.0%**	**165**	**100.0%**	**694**	**100.0%**
Age ineligible[i]	1	0.2%	0	0.0%	1	0.2%	0	0.0%	0	0.0%	0	0.0%	1	0.1%
Gone for duration of study	0	0.0%	0	0.0%	0	0.0%	1	0.8%	0	0.0%	1	0.6%	1	0.1%
Incapable (mental/physical impairment)	0	0.0%	1	1.0%	1	0.2%	0	0.0%	1	1.0%	1	0.6%	2	0.3%
Screening incomplete eligibility unknown	67	15.6%	17	17.0%	84	15.9%	27	22.0%	4	9.5%	31	18.8%	115	16.6%
Ineligible (previous breast cancer)	11	2.6%	0	0.0%	11	2.1%	3	2.4%	1	2.4%	4	2.4%	15	2.2%
Quota met	72	16.8%	2	2.0%	74	14.0%	4	3.3%	1	2.4%	5	3.0%	79	11.4%

[a]Physically or mental impaired individuals who cannot because of this limitation participate; households in which a resident cannot be accessed to determine eligibility; respondent or informant language barriers (other than Polish);

[b]Households or individuals that do not meet the study requirements, businesses, other non-residences such as group homes, military barracks, and retirement centers.

[c]Households with 12 no answers, answering machines, busy signals without human contact.

[d]Disconnected or disabled phone numbers, numbers with repeated (at least 5) technical problems (i.e. circuits, temporarily not in service).

[e]The sample was stratified by age and density with a targeted number of controls for each cell. Once the targeted number of controls was reached in the cell, further recruitment was closed.

Refusal	11	2.6%	5	5.0%	16	3.0%	2	1.6%	1	2.4%	3	1.8%	19	2.7%
Screened as eligible	**267**	**62.2%**	**75**	**75%**	**342**	**64.7%**	**83**	**67.5%**	**34**	**81.0%**	**117**	**70.9%**	**459**	**66.1%**
**Enrolled in Study**	**267**		**75**		**342**		**83**		**34**		**117**		**459**	

[f]Informant or respondent' refusal to participate, either in the screening process or in the research.
[g]Households with less than 12 no answers, answering machines, busy signals. No human contact made.
[h]Households or individuals initially identified as Polish, unable to complete screening during the data collection time period
iDuring final screening process determined respondent was over study eligible age criteria

Table S2. Disposition of Identified Controls amongst Polish-born women residing in Cook County IL or in the Detroit Metropolitan Area, MI

	Cook County		Detroit Metropolitan Area		Total	
**Disposition**	**N**	**% of All Controls**	**N**	**% of All Controls**	**N**	**% of All Controls**
Identified as eligible by IPPSR	**350**		**109**		**459**	
Identified as eligible by Gordon Research[a]	**8**					
**Total Identified as eligible controls**	**358**		**109**	**100.0**	**467**	**100.0**
Ineligible-upon interviewer-contact	42	11.7	9	8.2	51	11.0
Cell quota met	42	11.7	-		42	9.0
Not Polish Born	-		6	5.5	6	1.3
Age	-		2	1.8	2	0.4
Not a resident of USA	-		1	0.9	1	0.2
**Sub-Total of identified**	**316**	**88.3**	**100**	**91.8**	**416**	**89.1**
Non-contacts	35	9.8	-		35	7.5
No action	20	5.6	-		20	4.3
No answer to phone contact	1	0.3	-		1	0.2
Answering machine/phone busy	1	0.3	-		1	0.2
Call back unsuccessful	4	1.1	-		4	0.9
Appointment pending	1	0.3	-		1	0.2
Telephone broken appointment	2	0.6	-		2	0.4
Telephone temporary refusal	6	1.7	-		6	1.3
**Sub-Total of identified**	**281**	**78.5**	**100**	**91.8**	**381**	**81.6**
Non-Interview	48	13.4	24	22.0	72	15.4
Diseased	1	0.3	-		1	0.2
Final unable to locate	9	2.5	1	0.9	10	2.1
Final refusal	31	8.7	20	18.3	51	10.9
R. too ill/Handicapped	1	0.3	2	1.8	3	0.6
Unavailable-entire field period	5	1.4	-		5	1.1
Other non-interview	1	0.3	1	0.9	2	0.4
**Sub-Total of identified**	**233**	**65.1**	**76**	**69.7**	**309**	**66.2**

	**N**	**% of All Controls**	**N**	**% of All Controls**	**N**	**% of All Controls**
**Control disclosed to be a case at interview**	6	1.7	1	0.9	7	1.5
**Completed face to face control interviews**	**227**	**63.4**	**75**	**68.8**	**302**	**64.7**

Cook County		Metropolitan Detroit		Total	
**Response Rate[b]**	**Cooperation Rate[c]**	**Response Rate**	**Cooperation Rate**	**Response Rate**	**Cooperation Rate**
**74.0**	**85.3**	**74.7**	**78.7**	**74.0**	**83.3**

[a] Gordon Research was the initial firm conducting control recruitment. Gordon Research used approximately 2,451 records to recruit the 8 controls. Because the company ceased operating shortly after starting recruitment, the disposition of the 2,451 cases is unknown and not accounted for in the numbers in Table S1.

[b] Response rates are calculated using the AAPOR formula RR4: (I+P)/((I+P) + (R+NC+O) + e(UH+UO)), (**I**nterviews **+** **P**artial **I**nterviews)/(**I**nterviews + **P**artial **I**nterviews) + (**R**efusals + **N**on-**C**ontact + **O**ther**)** + **e** (**U**nknown **H**ousehold)+(**U**nknown **O**ther). **e** is the estimated proportion of cases of unknown eligibility that are eligible.
[c]Cooperation rates are calculated using the AAPOR formula CR4: (I+P)/((I+P)+R)), (**I**nterviews + **P**artial **I**nterviews)/(**I**nterviews + **P**artial **I**nterviews) + (**R**efusals)

Table S3. Identification and Disposition of Cancer Cases amongst Polish-born women residing in Cook County IL or in the Detroit Metropolitan Area, MI

	Cook County (ISCR)		Metropolitan Detroit (KCI)		Total All Sites	
**Category**	**N**	**% of Category A**	**N**	**% of Category A**	**N**	**% of Category A**
**A. Screened for eligibility at ISCR/KCI**	3,341	100.0%	20,721	100.0%	24,062	100.0%
Ineligible[a]	313	9.4%	1,757	8.5%	2,070	8.6%
Unable to determine eligibility	1,008	30.2%	3,065	14.8%	4,073	16.9%
Not Polish born	1,754	52.5%	6,120	29.5%	7,874	32.7%
Possible not Polish born[b]	-	-	9,716	46.9%	9,716	40.4%
**Polish born**	**266**	**6.5%**	**62**	**0.3%**	**328**	**1.4%**
	**N**	**% of Category B**	**N**	**% of Category B**	**N**	**% of Category B**
**B. Polish Born**	**266**	**100.0%**	**62**	**100.0%**	**328**	**100.0%**
**B1. Ineligible**	**52**	**100%**	**3**	**100.0%**	**55**	**100.0%**
Died	25	48.0%	2	66.7%	27	49.0%
Moved from study area	27	52.0%	1	33.3%	28	51.0%
**B2. Eligibility Unknown**	**75**	**100.0%**	**2**	**100.0%**	**77**	**100.0%**
Unable to trace	35	46.7%	-	-	35	45.5%
Physician refusal	37	49,3%	-	-	37	48.0%
Unable to complete verification	3	4.0%	2	100.0%	5	6.5%
**B3. Eligible Polish born**	**139**	**100.0%**	**57**	**100.0%**	**196**	**100.0%**
Refused participation	16	11.5%	12	21.1%	28	14.3%
Incapable of participation	10	7.2%	5	8.8%	15	7.7%
**Recruited into study ISCR/KCI**	**113**	**81.3%**	**40**	**70.2%**	**153**	**78.1%**
**B4. Completed interview**	**105**	92.9%	**35**	87.5%	**140**	91.5%
**Non-Interviews**	**8**	**7.1%**	**5**	**12.5%**	**13**	**8.5%**
**B4.1. Non-Interviews**	**8**	**100.0%**	**5**	**100.0%**	**13**	**100.0%**
Deceased	1	13%	-		1	8%
Duplicate	2	25%	-		2	15%
No follow-up with recruiter	2	25%	5	100.0%	7	54%
Refused	2	25%	-		2	15%
Unreachable	1	13%	-		1	8%
**B4. Additional Recruitment**	**11**	**100.0%**	**1**	**100.0%**	**12**	**100.0%**
Control disclosed had BC at interview	6	54.5%	1	100.0%	7	58.3%
Late recruitment[c]	5	45.5%	-		5	41.7%
	**N**	**% of Category C**	**N**	**% of Category C**	**N**	**% of Category C**
**C. Final Completed Interviews**	**116**	**100.0%**	**36**	**100.0%**	**152**	**100.0%**

[a]Due to age, sex, race, death prior to contact, diagnosis date, moved out of the study area.

[b]Possible not Polish name or foreign sounding name other than Polish

[c]After NORC's contract with PWHS was finalized, 5 more cases were interviewed by the same interviewer, now employed by MSU

[d]14 had an ineligible date of diagnosis, 4 resided outside study areas, and 3 were residents of census tracts not included in the sampling

Final Verification[d]	-15	12.9%	-6	16.7%	-21[d]	13.8%
**Final Analytic Data Set**	**101**	**87.1%**	**30**	**83.3%**	**131**	**84.2%**
**D. Response rates[e]**	**76.6%**		**57.1%**		**70.4%**	
**E. Cooperation rates[e]**	**85.4%**		**75.0%**		**82.4%**	

frame for controls

[e]Response rates are calculated using the AAPOR formula RR4: (I+P)/((I+P) + (R+NC+O) + e(UH+UO)), (Interviews + Partial Interviews)/(Interviews + Partial Interviews) + (Refusals + Non-Contact + Other) + e (Unknown Household)+(Unknown Other). e is the estimated proportion of cases of unknown eligibility that are eligible.

[e]Cooperation rates are calculated using the AAPOR formula CR4: (I+P)/((I+P)+R)), (Interviews + Partial Interviews)/(Interviews + Partial Interviews) + (Refusals)
